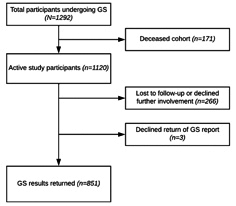# Findings from comprehensive genome sequencing in the Canadian population: Results from the GENCOV Study

**DOI:** 10.1016/j.gimo.2026.104372

**Published:** 2026-02-04

**Authors:** Selina Casalino, Navneet Aujla, Erika Frangione, Radhika Mahajan, David Di Iorio, Chun Yiu Jordan Fung, Lochana Jayachandran, Georgia MacDonald, Gregory Morgan, Dawit Wolday, Juliet Young, Saranya Arnoldo, Erin Bearss, Alexandra Binnie, Bjug Borgundvaag, Sunakshi Chowdhary, Marc Clausen, Marc Dagher, Luke Devine, Brendan Dickson, Steven Marc Friedman, Anne-Claude Gingras, Lee W. Goneau, Zeeshan Khan, Elisa Lapadula, Tony Mazzulli, Allison McGeer, Shelley L. McLeod, Chloe Mighton, Trevor J. Pugh, David Richardson, Stephen W. Scherer, Jared Simpson, Seth Stern, Ahmed Taher, Lisa J. Strug, Yvonne Bombard, Hanna Faghfoury, Elena Greenfeld, Limin Hao, Matthew Lebo, William Lane, Abdul Noor, Jennifer Taher, Jordan Lerner-Ellis, HostSeq Implementation Committee, Bartha Knoppers, Lisa J. Strug, Mark Lathrop, Naveed Aziz, Stephen W. Scherer, Steven Jones, Stuart Turvey

**Affiliations:** 1 Mount Sinai Hospital, Sinai Health, Toronto, ON, Canada; 2 Lunenfeld-Tanenbaum Research Institute, Sinai Health, Toronto, ON, Canada; 3 University of Toronto, Toronto, ON, Canada; 4 William Osler Health System, Brampton, ON, Canada; 5 Schwartz/Reisman Emergency Medicine Institute, Sinai Health, Toronto, ON, Canada; 6 University Health Network, Toronto, ON, Canada; 7 Genomics and Health Services Research Program, Unity Health Toronto, Toronto, ON, Canada; 8 Women’s College Hospital, Toronto, ON, Canada; 9 Dynacare Medical Laboratories, Brampton, ON, Canada; 10 Mackenzie Health, Richmond Hill, ON, Canada; 11 Ontario Institute for Cancer Research, Toronto, ON, Canada; 12 The Centre for Applied Genomics, Genetics and Genome Biology, The Hospital for Sick Children, Toronto, ON, Canada; 13 McLaughlin Centre and Department of Molecular Genetics, University of Toronto, Toronto, ON, Canada; 14 Laboratory for Molecular Medicine, Partners Personalized Medicine, Cambridge, MA; 15 Harvard Medical School and Brigham and Women’s Hospital, Boston, MA

**Keywords:** Comprehensive genome reporting, Genome sequencing, Genomic screening, Opportunistic screening, Secondary findings

## Abstract

**Purpose:**

Opportunistic genome sequencing (GS) allows for the return of findings to clinical and research cohorts. We report on comprehensive GS results from the GENCOV study in Ontario, Canada.

**Methods:**

GS data were analyzed for clinically significant variants associated with monogenic disease and carrier status for autosomal recessive and X-linked conditions, pharmacogenomic variation, polygenic risk scores for common conditions, human leukocyte antigen and blood group genotypes, and genetic ancestry. GS results were summarized using descriptive statistics.

**Results:**

GS was completed on 1292 participants; 53% were female, 53% were 18 to 39 years old, and 816 (63%) were estimated to have European genetic ancestry. All (100%) had a variant associated with drug metabolism, 845 (65%) with increased polygenic risk scores, 735 (57%) with a risk-associated human leukocyte antigen genotype, and 857 (69%) and 91 (7%) with a rare red blood cell and/or platelet antigen, respectively. Of 851 who received reports, 261 (31%) had a variant associated with monogenic disease (178 or 21% were considered medically actionable) and 782 (92%) had at least one variant associated with carrier status.

**Conclusion:**

Opportunistic GS demonstrated that many individuals harbor GS findings impacting their health, illustrating the potential of GS to inform personalized and proactive health care for Canadians.

## Introduction

Genomic technologies are rapidly advancing the field of genomic and precision medicine. Genome sequencing (GS) is increasingly being used for clinical diagnosis, as well as in research and opportunistic screening, wherein GS data initially generated for one purpose may be utilized for other secondary analyses.^1^, ^2^, ^3^ For instance, the HostSeq initiative utilized GS data collected from several studies across Canada to characterize genetic factors affecting COVID-19 infection and outcomes.^4^ One of these studies, called GENCOV, opportunistically returned findings related to hereditary disease risks identified from GS to inform individuals’ future health in addition to contributing GS data to HostSeq.^5^^,^^6^ In this context, GS may be used preventatively as a screening tool to identify risk for hereditary conditions that have established management or treatment recommendations for early detection and disease prevention, or allow for individuals to plan accordingly for their futures (eg, financial or family planning).^3^ Beyond findings related to monogenic disease, GS data have been used to identify pharmacogenomics variants related to drug metabolism,^7^^,^^8^ blood groups,^9^ human leukocyte antigen (HLA) genotypes,^10^ genetic ancestry,^11^ and polygenic risk scores (PRS) for common health conditions.^12^ Therefore, there is a wealth of information to be gathered from GS that, taken together, has the ability to inform proactive, personalized health care and precision medicine.

Several large-scale sequencing studies have been published describing return of GS findings to study participants; however, the return of results are generally limited to those considered to be medically actionable,^13^, ^14^, ^15^ for instance, as defined by the American College of Medical Genetics (ACMG).^16^ In addition, large-scale sequencing studies offering return of GS findings to study participants often include cohorts selected for specific disease status or clinical phenotypes, which are not representative of the general public.^14^^,^^17^^,^^18^ There are some sequencing projects and initiatives across North America offering return of findings beyond those considered to be medically actionable to research participants, including in the context of predispositional or opportunistic GS.^8^^,^^19^, ^20^, ^21^, ^22^, ^23^, ^24^, ^25^ However, these projects differ in sequencing technology (eg, genome versus exome sequencing), the type of information returned (eg, different composition of gene panels, pharmacogenomics findings, and complex traits), as well as whom results are returned to (eg, healthy individuals versus disease-selected cohorts).

To our knowledge, there are no published studies reporting the yield of clinically significant variation in addition to other, broad health-related findings from GS, including blood group, PRS, pharmacogenomics variants, HLA genotype and genetic ancestry, in a large cohort sampled from the general public. Here, we describe the yield of comprehensive GS findings across a broad range of categories related to personal health and disease risks from a large, ostensible healthy population-based cohort recruited through the GENCOV study. In addition, we review how these results compare with previously published large-scale sequencing studies, as well as discuss implications for use of GS as a predispositional screening tool in the general Canadian population.

## Materials and Methods

A comprehensive bioinformatics pipeline for genome reporting through the GENCOV study has been previously published.^26^ In brief, the GENCOV study aimed to identify serological, viral, and host genetic factors impacting SARS-CoV-2 (COVID-19) susceptibility and outcomes^5^ as part of the HostSeq Initiative.^4^ Adults (18 years of age or older) from the general public in the Greater Toronto Area (GTA) with a previous COVID-19 diagnosis (either by SARS-CoV-2 polymerase chain reaction or rapid antigen test) underwent GS (*N* = 1292). One-hundred and seventy-two (*n* = 172) participants were part of a deceased cohort (ie, as a result of their COVID-19 infection) and therefore unable to consent to return of GS results. The remaining active, live-study participants (*n* = 1120) were able to opt in to receive a broad range of findings from GS, including: risk for monogenic conditions, carrier status, pharmacogenomics variants associated with drug metabolism, PRS for common health conditions (atrial fibrillation, coronary artery disease, type 2 diabetes, colorectal cancer, breast cancer in females, and prostate cancer in males), HLA genotype, blood group genotype, and genetic ancestry estimation.^6^^,^^27^ The rationale was to be as comprehensive in the reporting as possible. In addition, given that GS was performed in the context of a COVID-19 study, certain data were analyzed and returned given the published relationship to COVID-19. For instance, *ABO* (HGNC:79) blood group has been linked to COVID-19 susceptibility, as well as protection against infection.^28^^,^^29^ In addition, data suggest that individuals of certain genetic ancestries or ethnicities have a greater risk of COVID-19 infection.^30^

Pretest counseling was provided using a genomic counseling model (GCM) consisting of a Genetic Counselor-led educational Zoom webinar lasting approximately 30 to 60 minutes and the Genetics Adviser evidence-based digital tool designed to support the genetic service pathway by providing education and exploring individual preferences and values.^27^^,^^31^ Group webinars provided additional information on possible findings from GS, considerations (eg, genetic discrimination, impact on family members), testing limitations, and general information about the GENCOV study. Participants were also able to ask anonymous questions at the end of the webinar. Preferences for GS findings were confirmed and recorded by the study Genetic Counselor after completion of the GCM. Results were returned to participants through a comprehensive genome report and summary letter based on preferences.^27^ Individuals with a pathogenic or likely pathogenic (P/LP) variant associated with personal risk for monogenic disease (eg, heterozygous for a P/LP variant in a gene associated with an autosomal dominant condition) were referred for clinical counseling, validation testing, and follow-up with a certified Genetic Counselor and Medical Geneticist,^6^ with the exception of those with common, high frequency P/LP variants in genes with reduced penetrance and often asymptomatic presentation, including *FLG* (HGNC:3748) and *F5* (HGNC:3542) Leiden, for which a summary letter with general recommendations were provided as described above. Likewise, findings that were not considered to be clinically or medically actionable (ie, without established clinical screening or management recommendations), as well as variants related to carrier status were not routinely sent for clinical validation and this limitation was explicitly indicated in both the GS reports and summary letters.

GS was performed using next-generation sequencing on the Illumina NovaSeq 6000 platform at The Center for Applied Genomics (TCAG) at SickKids Hospital in Toronto, ON, Canada. Data processing and analysis were performed using local analytic pipelines that used Genome Analysis Toolkit (GATK) best practices (GATK 3.7; GRCh38/hg38 alignment: GCA_000001405.15) and the Franklin Genoox Platform (genoox.com; GRCh37/hg19 alignment: https://ftp-trace.ncbi.nih.gov/1000genomes/ftp/technical/reference/human_g1k_v37.fasta.gz).^6^ Copy-number variants (CNVs) were called with an estimation by read depth with single-nucleotide variants (ERDS) and CNVator based workflow for 272 samples (GRCh37/hg19 alignment)^32^ and with DRAGEN software v3.8.4 for 1020 samples (GRCh38/hg38 alignment). Annotated CNVs received directly from the TCAG were used for this study. Custom filters were applied to identify single-nucleotide variants (SNV) for further analysis based on (1) aggregated or internal allele frequencies of ≤ 5% and (2) in genes with established gene-disease relationships, or (3) prior pathogenic or likely pathogenic (P/LP) classification in ClinVar or by the Genoox by Franklin algorithm.^6^ CNVs were filtered and identified for further analysis based on the presence of known exonic and/or OMIM-morbid genes in the region and haplo- or triplo-sensitivity scores. CNVs with >90% overlap with known benign, polymorphic CNV regions were excluded. CNV size (ie, >20Kb) and frequency (ie, <1%) were considered in the review process. SNVs and CNVs were filtered further by quality parameters (eg, confidence of call), availability of published literature, evidence from ClinVar submissions, validity of gene-disease relationships, and participant preferences.^6^ Carrier status for Spinal Muscular Atrophy (SMA; MIMs: 253300, 253550, 253400, and 271150) was detected using SMNCopyNumberCaller.^33^ Variants were subsequently analyzed and classified following the ACMG criteria and guidelines for SNVs and CNVs^34^^,^^35^ using in-house analytical pipelines and software tools. A list of 213 medically actionable genes for the GENCOV study (Supplemental File A) was developed according to ACMG secondary findings (SF) list,^17^ The Clinical Genome Resource by ClinGen,^36^ Reble et al^37^ (2021), and expert review (eg, by Genetic Counselors, Clinical and Molecular Geneticists).^6^ Clinically significant variants are those classified as P/LP by the study team. Variants classified as uncertain significance were not reported. Repeat expansions, mitochondrial variants, and CNVs associated with carrier status for autosomal recessive conditions were not routinely reported. Variants considered to be risk factors were reported as clinically significant at the discretion of the study team depending on factors such as medical actionability and impact on screening recommendations. Heterozygous P/LP variants associated with autosomal dominant conditions, homozygous or compound heterozygous P/LP variants associated with autosomal recessive conditions, and hemizygous P/LP variants associated with X-linked conditions (ie, in male participants) were categorized as medically actionable if identified in a gene included on the medically actionable gene list (Supplemental File A). All other variants were considered nonactionable findings associated with rare Mendelian disease. Heterozygous variants identified in genes associated with autosomal recessive or X-linked inheritance were considered carrier status findings.

Pharmacogenomic variants were detected using Stargazer^7^ and reported based on The Pharmacogenomics Knowledge Base (PharmGKB) recommendations and Pharmacogenetics Implementation Consortium (CPIC) dosage guidelines.^26^ Variants with CPIC level A and PharmGKB level 1A evidence with moderate or high evidence related to prescribing guidance for an associated medication were included in the report. PRS scores were calculated using existing algorithms previously described by Hao et al.^12^ HLA-VBSeq v2 software^10^ was used to estimate the most probable HLA types from GS data. The top-called alleles for the 7 major HLA loci were then compared with an in-house database containing currently known HLA disease associations based on review of the literature.^26^ Blood group genotyping from GS data was performed as previously described.^9^ Large-scale continental genetic ancestry was estimated by comparing patient genotypes to those from the HapMap3 reference data set.^26^^,^^38^ ADMIXTURE was used to output estimated patient ancestry against the reference data set after filtration.^11^ Demographic information including sex and date of birth were collected from clinical chart review and/or patient-facing surveys.^6^^,^^27^ Results were summarized using descriptive statistics.

## Results

A total of 1292 individuals were sequenced by GS as part of the GENCOV study, of which, 172 were deceased and excluded from return of results. The remaining 1120 participants were active study participants, of which 266 were lost to follow-up or declined further involvement, and 3 declined return of a GS report post-GCM (Supplemental Figure 1). Therefore, a total of 851 participants consented to return of GS results through a comprehensive genome report as described above. Six-hundred and eighty-eight (688, 53%) of total study participants (*N* = 1292) were female, 604 (47%) were male, 206 (16%) were 18 to 29 years of age, 245 (19%) were 30 to 39 years of age, 228 (18%) were 40 to 49 years of age, 229 (18%) were 50 to 59 years of age, 175 (14%) were 60 to 69 years of age, 108 (8%) were 70 to 79 years of age, and 97 (8%) were 80 years of age or older. Age was unknown or not collected for 4 (<1%) individuals. The majority of individuals’ estimated genetic ancestry aligned with the following HapMap3 reference data sets: 496 (38%) Toscani in Italia, 320 (25%) Utah residents with Northern and Western European ancestry, 205 (16%) Gujarati Indians in Houston, Texas, 106 (8%) Chinese in Metropolitan Denver, Colorado, 79 (6%) Yoruba in Ibadan, Nigeria, 54 (4%) Mexican ancestry in Los Angeles, California, 19 (1%) Han Chinese in Beijing, China, 7 (1%) Japanese in Tokyo, Japan, 4 (<1%) Maasai in Kinyawa, Kenya, and 2 (<1%) Luhya in Webuye, Kenya (Table 1).

**Table 1:** Demographic information

Finding	Number of Individuals (%)
Sex	
Female	688 (53)
Male	604 (47)
Age	
18-29	206 (16)
30-39	245 (19)
40-49	228 (18)
50-59	229 (18)
60-69	175 (14)
70-79	108 (8)
80+	97 (8)
Unknown	4 (<1)
Estimated Genetic Ancestry	
Toscani in Italia (TSI)	496 (38)
Utah residents with Northern and Western European ancestry (CEU)	320 (25)
Gujarati Indians in Houston, Texas (GIH)	205 (16)
Chinese in Metropolitan Denver, Colorado (CHD)	106 (8)
Yoruba in Ibadan, Nigeria (YRI)	79 (6)
Mexican ancestry in Los Angeles, California (MXL)	54 (4)
Han Chinese in Beijing, China (CHB)	19 (1)
Japanese in Tokyo, Japan (JPT)	7 (1)
Maasai in Kinyawa, Kenya (MKK)	4 (<1)
Luhya in Webuye, Kenya (LWK)	2 (<1)

Total *N* = 1292.

### Monogenic disease risks and carrier status

**Figure 1:**
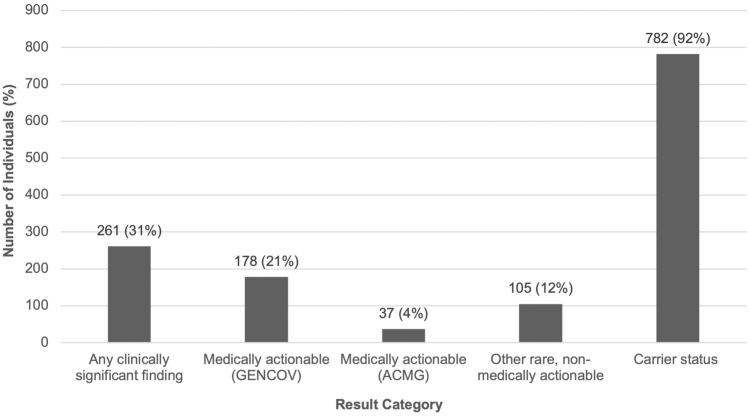
**A total of 851 study participants had genome reports returned to them from the GENCOV study.** Two-hundred and sixty-one (261, 31%) participants had at least 1 finding considered to be clinically significant with impact on their personal health, 178 (21%) had a finding included on the GENCOV medically actionable panel, 37 (4%) had a finding considered to be reportable based on current ACMG recommendations (SF list v3.2), 105 (12%) had a finding associated with a rare Mendelian disease considered not to be medically actionable, and 782 (92%) had a finding associated with carrier status with implications for reproductive planning. Please note that it is possible for 1 participant to have findings from multiple categories.

**Figure 2:**
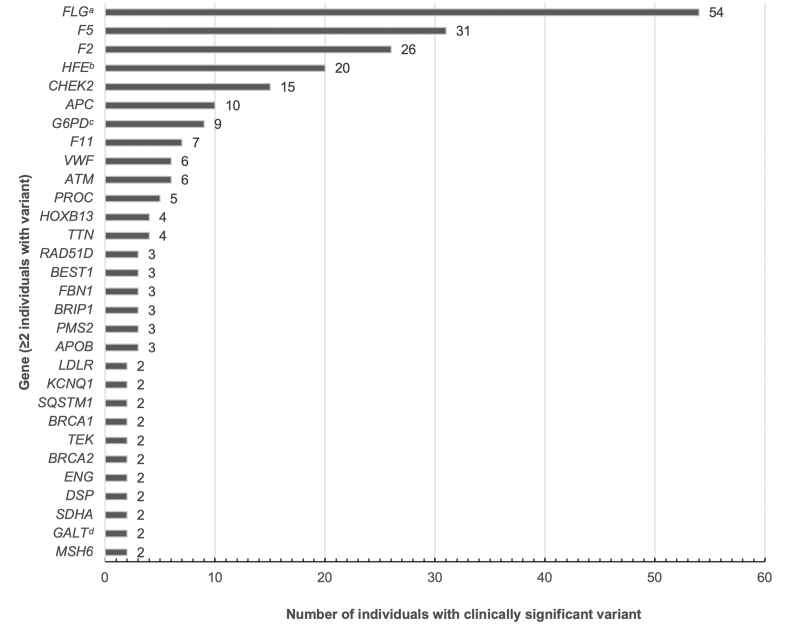
**Individuals with a clinically significant variant identified in genes associated with personal disease or health risk(s).** Because of the large number of genes in which a single variant was reported, only genes with clinically significant variants identified in 2 or more participants are included here; all genes in which clinically significant variants were identified can be found in Supplemental File B. ^a^Three individuals had 2 variants reported (compound heterozygous or homozygous). ^b^Homozygous or compound heterozygous. ^c^One homozygous female. ^d^Homozygous for Duarte variant.

Of those who received comprehensive genome reports (*n* = 851), 261 (31%) unique individuals had at least 1 clinically significant (P/LP) variant associated with personal risk(s) for a monogenic condition, including autosomal dominant actionable and nonactionable, rare Mendelian disease (Figure 1). There were 209 (25%) individuals with 1 variant, 42 (5%) with 2, 8 (1%) with 3, and 2 (<1%) with 4. Variants were identified most frequently in *FLG* (HGNC:3748) in 54 (6%) individuals (3 compound heterozygous or homozygous), followed by *F5* (HGNC:3542) (31, 4%), *F2* (HGNC:3535) (26, 3%), and *HFE* (HGNC:4886) (20 homozygous or compound heterozygous individuals, 3%) (Figure 2). Excluding more common variants found in the aforementioned genes (eg, *F5* [HGNC:3542] Leiden variant NM_000130.4:c.1601G>A p.(Arg534Gln); NC_000001.10:g.169519049=, *F2* [HGNC:3535] prothrombin variant NM_000506.5:c.∗97G>A; NC_000011.9:g.46761055G>A, and *FLG* [HGNC:3748] NM_002016.2:c.1501C>T p.(Arg501∗); NC_000001.10:g.152285861G>A variant associated with ichthyosis vulgaris [MIM: 146700]), the highest frequency of P/LP variants were identified in *CHEK2* (HGNC:16627) in 15 (2% [13 with the NM_007194.4 :c.470T>C p.(Ile157Thr); NC_000022.10:g.29121087A>G risk factor variant]) individuals, *APC* (HGNC:583) (10, 1% [8 with the NM_000038.6:c.3920T>A p.(Ile1307Lys); NC_000005.9:g.112175211T>A risk factor variant]), *G6PD* (HGNC:4057) (9, 1%), and *F11* (HGNC:3529) (7, 1%) (Figure 2). One-hundred and seventy-eight (178, 21%) individuals had a clinically significant variant in a gene included on the GENCOV medically actionable panel. In addition to the genes listed above, P/LP variants considered to be medically actionable by the study team were identified in *ATM* (HGNC:795) (6, 1%), *VWF* (HGNC:12726) (6, 1%), *PROC* (HGNC:9451) (5, 1%)*, TTN* (HGNC:12403) (4, <1%)*,* and *HOXB13* (HGNC:5112) (4, <1%). Notably, 37 (4%) individuals had a finding that would have been reported based on the current ACMG SF list (v3.2).^16^ In addition, a total of 5 (1%) individuals had a P/LP CNV associated with personal disease risk(s). A complete list of reported genes and variants are listed in Supplemental File B.

**Figure 3:**
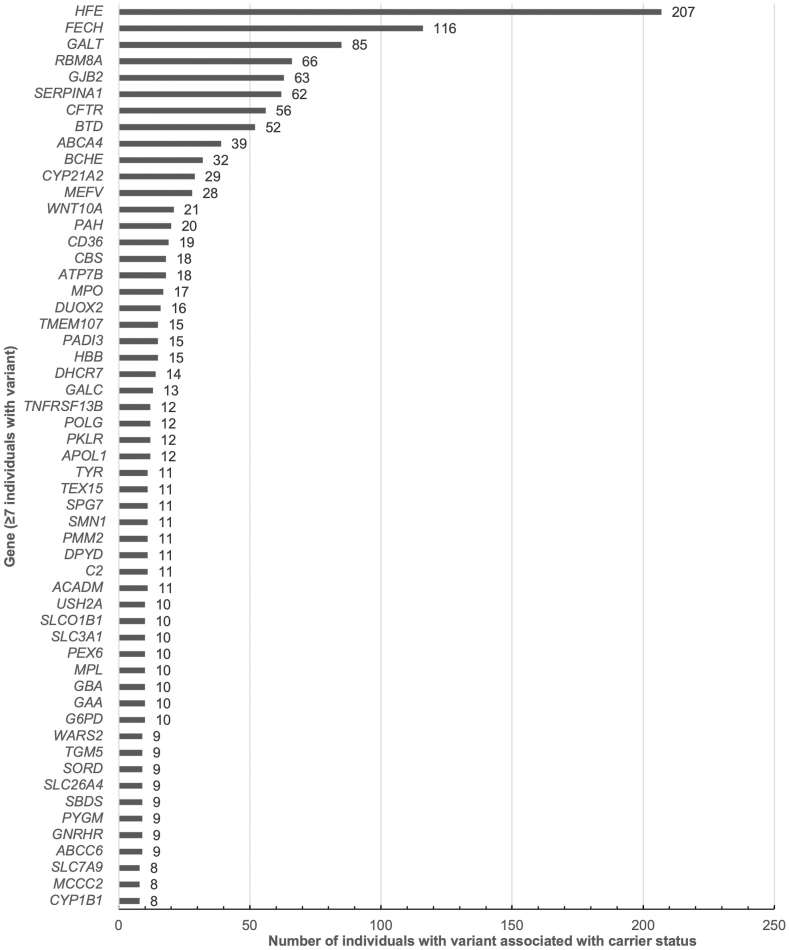
**Individuals with variants identified in genes associated with carrier status.** Because of the large number of genes in which carrier status variants were reported, only genes in which 8 or more participants had variants reported are included here; all carrier status genes can be found in Supplemental File C.

Seven-hundred and eighty-two (782, 92%) individuals had at least 1 P/LP variant associated with carrier status (Figure 1), with an average of ∼4 variants per person. There were 66 (8%) individuals with 1 clinically significant variant associated with carrier status, 116 (15%) with 2, 168 (21%) with 3, 185 (24%) with 4, 102 (13%) with 5, 69 (9%) with 6, 39 (5%) with 7, and 37 (5%) with 8 or more. Variants associated with carrier status were identified most frequently in *HFE* (HGNC:4886) in 207 (24%) unique individuals, followed by *FECH* (HGNC:3647) (116, 14%), *GALT* (HGNC:4135) (85, 10% [81 with Duarte variant]), *GJB2* (HGNC:4284) (63, 7%), and *SERPINA1* (HGNC:8941) (62, 7%) (Figure 3). In addition, 11 (1%) individuals were identified to have an *SMN1* (HGNC:11117) copy number of 1, consistent with carrier status for SMA (MIMs: 253300, 253550, 253400, and 271150). A complete list of genes with variants reported in the context of carrier status are listed in Supplemental File C.

### Pharmacogenomic variation

One-hundred percent (100%) of those who underwent GS (*N* = 1292) had at least 1 pharmacogenomic variant related to a significant metabolizer phenotype affecting recommendations for medication use and/or dosage, with an average of ∼5 variants per person (Table 2). The single most common pharmacogenomic variant identified was *CYP3A5* (HGNC:2638) ∗3/∗3 (rs776746) (observed in 929 or 72% of individuals), which is associated with being a poor metabolizer of Tacrolimus, an immunosuppressive drug that is given to prevent rejection after an organ transplant. A complete list of pharmacogenomics variants reported are listed in Supplemental File D.

**Table 2:** Summary of broad genome sequencing (GS) findings from the GENCOV study

Finding	Number of Individuals (%)
Pharmacogenomic variant	1292 (100)
Increased PRS	845 (65)
Atrial fibrillation	167 (13)
Coronary artery disease	185 (14)
Type 2 diabetes	201 (16)
Colorectal cancer	252 (20)
Breast cancer (females)	190 (15)
Prostate cancer (males)	227 (18)
Clinically significant *HLA* genotype	735 (57)
Ankylosing spondylitis	5 (<1)
Behçet’s disease	137 (11)
Celiac disease	931 (72)
Graves’ disease	21 (2)
Multiple sclerosis	60 (5)
Rheumatoid arthritis	8 (1)
Type 1 diabetes	21 (2)
Blood type^a^	
A	418 (34)
B	253 (20)
O	493 (40)
AB	71 (6)
Rh+	1087 (88)
Rh−	128 (10)
Rh+/-, weak/partial	20 (2)
Rare RBC antigen	857 (69)
Rare platelet antigen	91 (7)

Total *N* = 1292.

a Total *N* for blood group genotyping = 1235.

### Polygenic risk for common conditions

Eight-hundred and forty-five (845, 65%) of those who underwent GS (*N* = 1292) had an increased PRS (equivalent to a 2-fold increased risk or greater) for at least 1 common health condition (Table 2). Five-hundred and thirty-six (536, 41%) had an increased PRS for 1 condition, 248 (19%) for 2 conditions, 54 (4%) for 3 conditions, and 7 (1%) for 4 conditions. One-hundred and sixty-seven (167, 13%) individuals had an increased PRS for atrial fibrillation, 185 (14%) for coronary artery disease, 201 (16%) for type 2 diabetes, 252 (20%) for colorectal cancer, 190 (15%) females for breast cancer, and 227 (18%) males for prostate cancer.

### HLA genotyping

Seven-hundred and thirty-five (735, 57%) individuals who underwent GS (*N* = 1292) had at least 1 HLA genotype previously reported in the literature in association with an increased risk (odds ratio of ∼2 or greater) for an autoimmune condition, including 5 (<1%) with an increased risk for ankylosing spondylitis, 137 (11%) for Behçet’s disease, 931 (72%) for celiac disease, 21 (2%) for Graves’ disease, 60 (5%) for multiple sclerosis, 8 (1%) for rheumatoid arthritis, and 21 (2%) for type 1 diabetes (Table 2). The most commonly observed HLA genotype was *DQA1* (HGNC:4942) ∗05:05 × 1, which was identified in 377 (29%) individuals and has been reported previously in the literature as being associated with an increased risk for celiac disease.^39^ A complete list of HLA genotypes reported are listed in Supplemental File E.

### Blood group genotyping

*ABO* (HGNC:79) blood group and Rhesus (Rh) antigen were genotyped for 1235 individuals (Table 2). The most common *ABO* (HGNC:79) blood type observed was type O (493, 40%), followed by type A (418, 34%), type B (253, 20%), and type AB (71, 6%). One-thousand and eighty-seven (1087, 88%) individuals were Rh positive (Rh+), and 128 (10%) individuals were Rh negative (Rh−). The remaining 20 (2%) individuals had weak and/or partial Rh types. Overall, the most common blood type reported was O, RhD+ (433, 35%), followed by A, RhD+ (362, 29%), and B, RhD+ (224, 18%). Eight-hundred and fifty-seven (857, 69%) individuals had at least 1 rare red blood cell (RBC) antigen (eg, KDAS+, Jk(a+w), VS+), and 91 (7%) had at least 1 rare platelet antigen (eg, HPA-13bw+, HPA-1a-) (Supplemental File F).

## Discussion

One-thousand and ninety-two ostensibly healthy individuals previously diagnosed with COVID-19 underwent GS as part of the GENCOV study in Ontario, Canada for the purposes of identifying host genetic factors affecting COVID-19 infection susceptibility, severity, and outcomes.^5^ As a part of this study, participants were presented with the additional opportunity to receive comprehensive GS reports with a broad range of health-related findings. Results were returned to 851 individuals. All study participants had at least one GS result reported, including findings associated with personal risk for monogenic disease (31%), carrier status (92%), drug metabolism (100%), increased PRS for common health conditions (65%), significant HLA (57%), and rare blood group antigen (77%) genotype.

Our reported yield of clinically significant findings associated with personal risk for monogenic, including actionable and nonactionable, disease (31%) is in line with previously reported yields of 21% to 35%.^19^^,^^21^, ^22^, ^23^ Higher yield is likely accounted for by higher frequency P/LP variants identified in genes associated with conditions exhibiting reduced penetrance or mild symptoms. For example, *FLG* (HGNC:3748)-associated ichthyosis vulgaris (MIM: 146700) or *F5* (HGNC:3542) Factor V Leiden deficiency (MIM: 188055). Although these conditions exhibit reduced penetrance and variable symptom severity, we considered these findings significant because there are interventions or recommendations available to mitigate or reduce risk for symptoms (eg, topical ointments, blood thinning medication). Our yield of ACMG actionable findings (4%) is in line with what has been previously reported across other genomic sequencing studies (1%-5.3%).^40^, ^41^, ^42^, ^43^, ^44^, ^45^, ^46^ Current ACMG guidelines recommend reporting only certain variants in genes included on the SF list. For instance, *HFE* (HGNC:4886) NM_000410.4: c.845G>A p.(Cys282Tyr); NC_000006.11: g.26093141G>A homozygotes, as well as P/LP variants in *APC* (HGNC:583). Therefore, not all *HFE* (HGNC:4886) homozygotes or compound heterozygotes nor reduced penetrance, risk factor variants in *APC* (HGNC:583) NM_000038.6: c.3920T>A p.(Ile1307Lys); NC_000005.9: g.112175211T>A would be reported. Here, we used a broader definition of “medically actionable” to develop a panel of 213 genes in which identification of a P/LP variant presented some level of clinical benefit or utility, including management, screening, or treatment recommendations, regardless of the severity, penetrance, or frequency of the condition; factors which limit the ACMG SF list.^16^ These findings were reported in the present study given the overall goal of analyzing and reporting on a broad GS findings.

The reported yield of carrier status findings (92%) is reflected by others across multiple studies (85%-94%).^19^^,^^21^^,^^23^^,^^47^ All individuals had a pharmacogenomic variant impacting drug metabolism, slightly greater than what has been previously reported 95%-97%.^19^^,^^23^ A smaller Canadian study (*N* = 56) called the Personal Genome Project opting to return pharmacogenomics variants to participants identified only 23% with risk for an adverse drug reaction.^22^ However, this study analyzed a small panel limited to 14 pharmacogenes. Here, we reported any variant with moderate or strong evidence for prescribing recommendations (ie, CPIC level A and PharmGKB level 1A). These may include clinical guidelines or drug label annotations with variant-specific dosing guidelines for a drug, which may need to be prescribed to an individual now or in the future for a particular clinical indication. Sixty-five percent (65%) of individuals had an increased polygenic risk for a common health condition, 57% had an HLA genotype previously reported in the literature associated with an increased risk of an autoimmune condition (ankylosing spondylitis, Behçet’s disease, celiac disease, Graves’ disease, multiple sclerosis, rheumatoid arthritis, and/or type 1 diabetes), and 77% had a rare blood antigen (RBC and or platelet antigen). Other studies have reported yields of 59% and 89% for increased PRS and risk alleles associated with common, multifactorial conditions.^19^^,^^23^ Disparities with previously reported yields may be accounted for by differing analysis or reporting methodology. For example, the number of genetic loci included in genome wide association studies, conditions included, and thresholds for reporting increased risk. Reported blood types are in line with the estimated distribution in the Canadian population, with the most common blood type being O, RhD+ in 39% of individuals, and the least common being AB, Rh− in 0.5% of individuals.^48^ We identified that 10% of individuals were Rh−, which has potential clinical implications. For example, individuals who are Rh− have a risk for Rh incompatibility and hemolytic disease of the newborn without the appropriate intervention (ie, Rh immune globulin). One study returning blood group genotypes from GS to participants in the MedSeq Project indicated that 31% of their cohort (*N* = 100) harbored a rare RBC and/or platelet antigen (<5% population frequency),^19^ compared with the 77% with a rare blood antigen in our cohort. Identification of rare and extended blood group genotypes are relevant for blood donation or transfusions, as well as organ transplantation. In Canada, individuals with rare blood types may register with the Canadian Blood Services as a desirable donor for those with rare blood types in need of blood product donations. We were not able to identify any GS studies returning HLA genotype results and associated disease risks to research participants.

Participants receiving GS as part of the GENCOV COVID-19 research study were representative of a sample of Canadians from the general population in Ontario. Recruitment for the GENCOV study occurred across several Hospitals and COVID-19 testing centers across the Greater Toronto Area, with the only inclusion criteria being over 18 years of age and diagnosed with COVID-19. Furthermore, individuals represented a diverse range of genetic ancestries, with 37% estimated as having a majority Chinese, Indian, Japanese, Mexican, or African genetic ancestry and the remaining 63% being majority European ancestry (Table 1). Our findings illustrate that 31% of these individuals had at least 1 clinically significant (P/LP or clinically relevant risk factor) variant. It is therefore possible that a significant proportion of the general Canadian population harbors a variant that may impact their personal health, as well as the health of their family members. This is of particular importance for conditions with well-established treatment, screening, and management recommendations, such as *TTN* (HGNC:12403)*-*associated dilated cardiomyopathy (MIM: 604145) and hereditary cancers.^16^ Even conditions not considered to be medically actionable, such as *BEST1* (HGNC:12703)-associated retinopathy (MIMs: 193220, 153700, 613194, and 193220), may be actionable in other ways to individuals. For instance, to make decisions to improve their quality of life or plan financially for the future.^49^

In many cases, individuals with actionable variation may lack a relevant personal or family history and otherwise remain undiagnosed due to lack of genetic testing eligibility or clinical diagnostic criteria. This is especially problematic for inherited conditions in which the presenting or initial symptom of the disease is severe or life-threatening, such as sudden cardiac arrest in inherited arrhythmias. GS, when used proactively as a screening tool, is able to identify these risks before complications arise so that individuals with a hereditary predisposition can be screened accordingly, ultimately reducing disease-related mortality. Population-based GS for high-risk conditions may also be cost-effective to the health care system. A recent health economic evaluation assessing the impact and cost-effectiveness of a national screening program in Australia demonstrated that GS as a screening tool would prevent thousands of cancers, coronary heart diseases, and deaths in a cost-effective manner for the public health care system.^50^ Likewise, we know that the majority of individuals (92%) have a heterozygous or hemizygous variant associated with carrier status for at least 1 hereditary condition that may affect reproductive planning and decision making. Up to 2% of couples are heterozygous for a P/LP variant associated with carrier status for the same autosomal recessive condition, with the proportion being even higher for consanguineous couples.^51^^,^^52^ Therefore, information gleaned from carrier screening is extremely helpful for couples who are family planning and may also be cost-effective, especially when considering averted severe disease costs.^53^ Indeed, findings from GS may have different utility depending on an individual’s stage of life or current circumstances.^23^ For instance, those who have not yet completed family planning may find greater utility in carrier status findings than those who have, as mentioned above.^23^ Likewise, pharmacogenic findings may present greater utility to those who are currently using prescribed medications.^54^ GS therefore has the potential to be used as an effective screening tool in the general population with varying utility for different individuals, especially as this technology becomes more widely available.

The use of GS as a population-based or opportunistic screening tool carries many ethical and practical considerations, including, but not limited to, decisional autonomy, the risk of genetic discrimination, psychological implications of findings, interpreting risks associated with conditions that have reduced penetrance and variable expressivity, unequal access to genetic testing services, and implementation at the national level.^3^^,^^55^ In Canada, the Genetic Non-Discrimination Act protects individuals from the use of genetic test results in areas outside of medical care and research, such as insurance and employment, with the goal of removing barriers to use of genetic testing services by the public.^56^ Globally, there are frameworks and policies aimed at ensuring the responsible and secure use of genomic and other health-related data^57^ as generation of large amounts of GS data, both clinically and in research settings, becomes more commonplace. The overarching goal of these laws and frameworks is to improve precision health care and advance genomic medicine while protecting human rights and privacy. An additional consideration is that rigorous health economic evaluations and cost-benefit analyses are required to determine the utility of implementing GS as a screening tool at scale beyond the current scope of use in research settings. Pilot studies or initiatives implementing screening with smaller panels, such as the one previous described in Australia,^50^ would be a reasonable and necessary step toward gathering evidence for or against the utility of broader screening. The findings presented in this study may be used as evidence to support the consideration of GS as a tool for precision health care in Canada. A hypothetical model for implementing GS at scale is to utilize GS as a first-line test, regardless of indication, for those seen at a Hospital or Medical Center. Theoretically, then, an individual could be sequenced once and their data could be opportunistically reanalyzed as needed throughout the lifespan.^58^ For example, to provide information related to carrier status when family planning or to identify potential drug interactions before prescribing a new medication (92% and up to 100% of individuals based on the present study, respectively). Digital tools and educational resources, such as the GCM and Genetics Adviser, would be essential for supporting the implementation of GS at scale and have been shown to aid in GS decision making by reducing decisional conflict and increasing knowledge of GS.^27^

There are several limitations to this study and reported GS findings. First, there are currently no established clinical recommendations for those with risks related to PRS findings or HLA genotype (eg, changes to screening frequency or initiation of medications), nor is there clear clinical utility of these findings at this time. Likewise, although we reported on pharmacogenomic variants with established evidence and recommendations for drug dosage and efficacy, there are many pharmacogenomic variants with limited evidence for impact on drug metabolism or warranting changes to medications. Variants in Mendelian genes associated with reduced penetrance, multiple forms of inheritance (eg, autosomal recessive and autosomal dominant, or semi-dominant inheritance), or moderately increased risk for conditions were included in reports and counted in our reported yields. For example, both monoallelic and biallelic variants in *F11* (HGNC:3529) have been reported in patients with factor XI deficiency (MIM: 612416), consistent with semidominant inheritance (CCID:004791). Another example is the c.470T>C p.(Ile157Thr) variant in *CHEK2* (HGNC:16627)*,* which is considered a cancer risk factor with low or reduced penetrance.^59^ We recognize that there are differing opinions on the clinical significance and actionability of these findings; however, they were included given the comprehensive scope of our genome reporting study. Second, variants were reported on a research basis and therefore were not confirmed by clinical testing, which is required in addition to a clinical genetics assessment before making any health-related or reproductive decisions. A comprehensive genetics assessment may also help to elucidate the significance of a variant in the context of clinical manifestations and personal and family history, for example, in cases in which variants are identified in genes associated with both autosomal dominant and autosomal recessive forms of inheritance (eg, *VWF* [HGNC:12726]*, BEST1* [HGNC:12703]). Additionally, not all types of genetic variation were detected or included based on our testing and reporting methodology. Third, genetic ancestry estimates were based on the HapMap3 data set and therefore was limited to 1.6 million common single-nucleotide variations (formerly single-nucleotide polymorphisms) in 1184 reference individuals from 11 global populations.^38^ It is also worth noting that genetic ancestry estimations may be different than someone’s self-reported ethnicity or race.^60^ Finally, we were unable to elucidate participants’ motivations for enrolling in the GENCOV study and opting to receive GS findings. For instance, it is possible that individuals opted to participate to receive a potential diagnosis from GS (eg, for a medical condition present in their family).^61^ These individuals may be more likely to have a P/LP variant related to monogenic disease risk, therefore inflating the observed yield of GS findings.

As part of our future work, we will compare estimated genetic ancestry with self-reported ethnicity, as well as compare the HapMap3 ancestry estimation with outputs generated using alternative reference data sets (eg, The 1000 Genomes Project). We will evaluate the impact, health outcomes, and changes to health-related behaviors as a result of receiving comprehensive GS findings through surveys and qualitative interviews. Qualitative interviews will also be effective for exploring individuals’ motivations for participating in the GENCOV study and opting to receive various GS results. We will also explore participants’ knowledge and understanding of results to adapt our GCM^27^ and reporting structure to better suit the needs of our study population. As part of the GENCOV study workflow,^6^ individuals identified to harbor a P/LP variant associated with personal risk for a monogenic, hereditary condition were referred to clinical services for validation of results, a complete genetics assessment (including comprehensive medical and family history review), and provided with a plan for follow-up and management (eg, specialist referrals, screening recommendations, and familial cascade testing). Future work will determine the number of individuals with findings consistent with reported medical and family history, the uptake and concordance of clinical confirmatory testing, downstream access to and impact on health care services, and compliance with clinical recommendations (eg, screening frequency, risk-reducing surgery).

### Conclusion

In conclusion, GS is a powerful screening tool that enables identification of a broad range of health-related findings that, when taken together, offer a comprehensive and personalized summary of an individual’s overall health risks. With GS being used more frequently in both the clinical and research settings, there is a wealth of data being generated that may be used opportunistically to inform future health risks and care. The GENCOV study presented an opportunity to evaluate the yield of GS in an ancestrally diverse, ostensibly healthy cohort, which illustrated that up to 31% of individuals harbor a clinically significant variant associated with personal risk for monogenic disease. Furthermore, many individuals had GS findings associated with an increased risk for complex, multifactorial disease (eg, autoimmune conditions, coronary artery disease, colon cancer), as well as variants associated with carrier status, drug metabolism, and rare blood group antigens affecting reproductive decisions, dosage recommendations, and blood transfusion or donation eligibility, respectively. Although additional research is needed to evaluate the utility and feasibility of GS as an opportunistic screening tool, future applications of GS should aim to align with the principles of proactive, personalized, and precision care to improve the overall health of Canadians.

## Data Availability

Deidentified data may be made available upon request to the corresponding author.

## Members of the HostSeq Implementation Committee

Bartha Knoppers, Lisa J. Strug, Mark Lathrop, Naveed Aziz, Stephen W. Scherer, Steven Jones, Stuart Turvey

## Conflict of Interest

Yvonne Bombard holds the Canada Research Chair in Genomics Health Services and Policy. Yvonne Bombard and Marc Clausen are cofounders of the Genetics Adviser. All other authors declare no conflicts of interest.

## Figures and Tables

**Supplementary Figure 1:**